# Pose Measurement Method and Experiments for High-Speed Rolling Targets in a Wind Tunnel

**DOI:** 10.3390/s141223933

**Published:** 2014-12-12

**Authors:** Zhenyuan Jia, Xin Ma, Wei Liu, Wenbo Lu, Xiao Li, Ling Chen, Zhengqu Wang, Xiaochun Cui

**Affiliations:** 1 Key Laboratory for Precision and Non-Traditional Machining Technology of the Ministry of Education, Dalian University of Technology, Dalian 116024, China; E-Mails: Jzyxy@dlut.edu.cn (Z.J.); maxin2000@mail.dlut.edu.cn (X.M.); me_lix@163.com (X.L.); 200961803@mail.dlut.edu.cn (L.C.); 2 AVIC Aerodynamics Research Institute, Shenyang 116024, China; E-Mails: F13py@163.com (W.L.); wzqnwpu@126.com (Z.W.); cui_xiaochun@163.com (X.C.)

**Keywords:** machine vision, pose measurement, high-speed motion, wind tunnel

## Abstract

High-precision wind tunnel simulation tests play an important role in aircraft design and manufacture. In this study, a high-speed pose vision measurement method is proposed for high-speed and rolling targets in a supersonic wind tunnel. To obtain images with high signal-to-noise ratio and avoid impacts on the aerodynamic shape of the rolling targets, a high-speed image acquisition method based on ultrathin retro-reflection markers is presented. Since markers are small-sized and some of them may be lost when the target is rolling, a novel markers layout with which markers are distributed evenly on the surface is proposed based on a spatial coding method to achieve highly accurate pose information. Additionally, a pose acquisition is carried out according to the mentioned markers layout after removing mismatching points by Case Deletion Diagnostics. Finally, experiments on measuring the pose parameters of high-speed targets in the laboratory and in a supersonic wind tunnel are conducted to verify the feasibility and effectiveness of the proposed method. Experimental results indicate that the position measurement precision is less than 0.16 mm, the pitching and yaw angle precision less than 0.132° and the roll angle precision 0.712°.

## 1. Introduction

The pose measurement method has been widely used in many research fields such as aerospace, robots and medical science. In some cases, the improper separation of stores carried by a high-speed aircraft, such as bombs, auxiliary fuel tank, torpedoes and missiles, will greatly impact the performance of the mother plane and even threaten the safety of the pilot [1,2]. Simulative store separation experiments in wind tunnels provide valuable reference for the layout design and release parameters of the store. However, since wind tunnel models are usually small-sized and move and rotate at high speeds, traditional measurements fail to correctly measure their pose information. Vision measurement has proved its advantages such as being non-contact and high precision and the ability for real-time measurement. Therefore, pose measurement in wind tunnels based on vision measurement is of great importance [3,4].

Numerous approaches for measuring the pose parameters of targets have been studied by many scholars and research institutions. Watzlavick *et al.* presented the Optotrak™ measurement system by Canada's Northern Digital Inc. (Waterloo, ON, Canada). Three precisely calibrated linear CCDs are used as displacement sensors [5]. The 3D coordinates of each marker can be obtained in real time by capturing the near-infrared light given out by the active luminous markers. The precision and resolution of the system can reach 0.1 mm RMS and 0.01 mm, respectively. It has been successfully applied to the aerodynamic elasticity and angle measurement of the wind tunnel models of both Boeing and the NASA Ames Research Center. However, its low sampling frequency and big volume of embedded markers cannot satisfy high-speed pose measurement demand of small volume objects. Rock *et al.* combined the Carrier-Phase Differential Global Positioning System (CDGPS) and a binocular vision system to identify ground target images [6]. They used color object identification technology to process images captured by the left and right cameras, and employed two different images to locate the target projection. Finally, the aircraft pose parameters can be estimated according to stereo triangulation. Though this method is simple in calculation, the wireless signals in a complex environment are susceptible to be disturbed, so the measurement data are not stable. NASA LaRC developed a pose measurement system based on binocular vision for the wind tunnel model [7–10]. The model images are captured continuously and synchronously by two CCD cameras through the observation window. The pose parameters of the wind tunnel model can be calculated after measuring the 3D centroid coordinates of circular markers. However, this method has disadvantages due to the hidden phenomenon when the model is rolling. Murray *et al.* employed an Optical Tracking System based on Particle Image Velocimetry (PIV) to obtain the pitch angle, angular velocity and related pose information of a high-speed target [11]. Though high-speed measurements can be achieved, they cannot get the rolling angle utilizing this system. Chen presented a pose measurement method for experimental model based on active vision in a wind tunnel [12]. This method transforms the direct pose measurement of the target into an indirect measurement of spots on the wall, which amplifies the variation of the information. Thus, the resolution of the measurement system can be indirectly improved, and the maximum error of displacement of the cooperating target is 0.46 mm. However, its low precision cannot satisfy pose measurement requirements of small volume high-speed target and high-speed rolling motion. Fan *et al.* proposed an attitude and position tracking system for indoor carriers based on an integrated navigation fusion of INS/UWB information strategy [13]. The two subsystems (INS and UWB) are integrated by a feedback correction method. Then, the data fusion of the two subsystems can be realized by an optimal comprehension and filtering strategy. Though the position and attitude information can be obtained in real time, when it comes to small targets with high-speed rolling motion in a wind tunnel, the UWB technology can hardly meet the measuring environment demands. In summary, scholars have made different kinds of investigations on innovations in pose measurement under wind tunnel conditions, but the pose measurement speed is low and the hidden phenomenon of cooperation markers in the rolling procedure is ignored.

In this paper, a pose measurement method for high-speed rolling targets in wind tunnels is presented. The remainder of this paper is organized as follows: Section 2 will detail the vision theory and image acquisition method; Section 3 will describe the marker layout on the surface of target and the image matching method; Section 4 will present the pose information acquisition method from the constructed markers' 3D coordinates; Section 5 will show the results obtained from a set of testing scenarios. Finally, the paper ends with conclusions about the whole pose measurement method and the results obtained.

## 2. Measurement Method and System for High-Speed and Rolling Targets

### 2.1. Binocular Vision Theory

**Figure 1.**
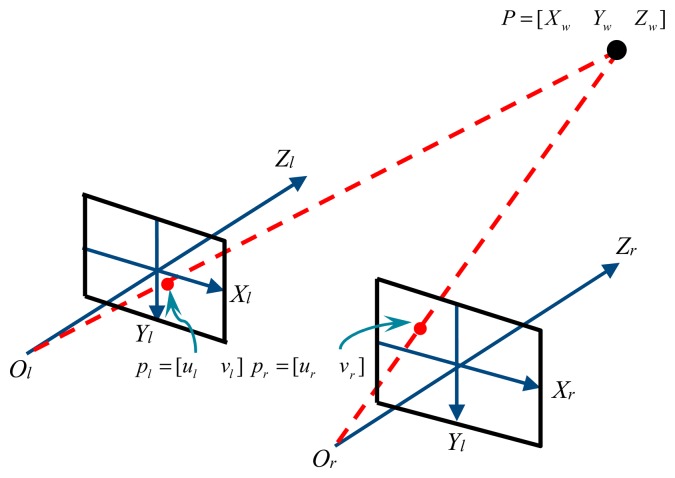
The principle of binocular stereo imaging.

Vision measurement focuses on how to obtain 3D information from 2D image information. Two types of coordinate systems (*i.e.*, image and global coordinate systems) are involved in transforming the 2D image coordinates obtained from the camera into 3D global coordinate information, as shown in Figure 1.

In this study, the pinhole model is taken as the imaging model. However, due to some defects occurred in the manufacture and installation procedures of composite lenses, image distortion is unavoidable, resulting in aberration between the real and theoretical image points. Therefore, we introduce a non-linear imaging model considering the impact of aberration. The projection equation of the non-linear imaging model can be given by:

(1) $$Z_{c}\left[\begin{array}{l} \hat{u}+\delta_{x}\\ \hat{v}+\delta_{y}\\ 1 \end{array}\right]=M_{1}\left[\begin{array}{cc} R & T\\ 0^{T} & 1 \end{array}\right]\;\left[\begin{array}{l} X_{w}\\ Y_{w}\\ Z_{w}\\ 1 \end{array}\right]$$

where *p*(*û v̂*) represents the real point caused by image distortion; (*δ_x_δ_y_*) stand for aberration, which are known already; (*X_w_Y_w_Z_w_*) represent the 3D coordinates of one point in the world coordinates; *Z_c_* is the scale factor; *M*_1_ represents the intrinsic parameter of the camera; rotation matrix *R* and translation matrix *T* are the extrinsic parameters.

Wide-angle lenses are used in our measurement system to obtain close shots with a large field of view, thus the main image distortion in our system is barrel distortion. To obtain a computation method with less complex and better stability, the first and second order distortion coefficients are employed in the aberration model, as shown below:

(2) $$\{\begin{array}{c} \delta_{x}=k_{1}\hat{u}\left({\hat{u}}^{2}+{\hat{v}}^{2}\right)+k_{2}\hat{u}{\left({\hat{u}}^{2}+{\hat{v}}^{2}\right)}^{2}\\ \delta_{y}=k_{1}\hat{v}\left({\hat{u}}^{2}+{\hat{v}}^{2}\right)+k_{2}\hat{v}{\left({\hat{u}}^{2}+{\hat{v}}^{2}\right)}^{2} \end{array}$$

where *k*_1_, *k*_2_ are the first and second coefficient of radial distortion.

**Figure 2.**
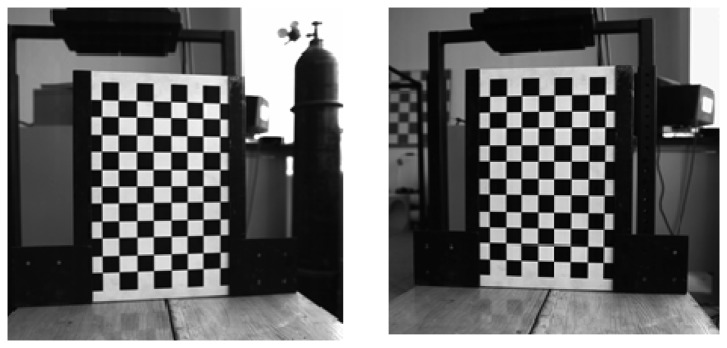
Calibration targets taken in different angles.

Camera calibration is the preliminary step for establishing an accurate relationship between the image and the real world. In this paper, Zhang's 2D calibration method is implemented to achieve a high precision calibration of the binocular vision measurement system with a wide field of 1 m × 1 m [14]. The calibration targets are shown in Figure 2.

### 2.2. Measurement Method

**Figure 3.**
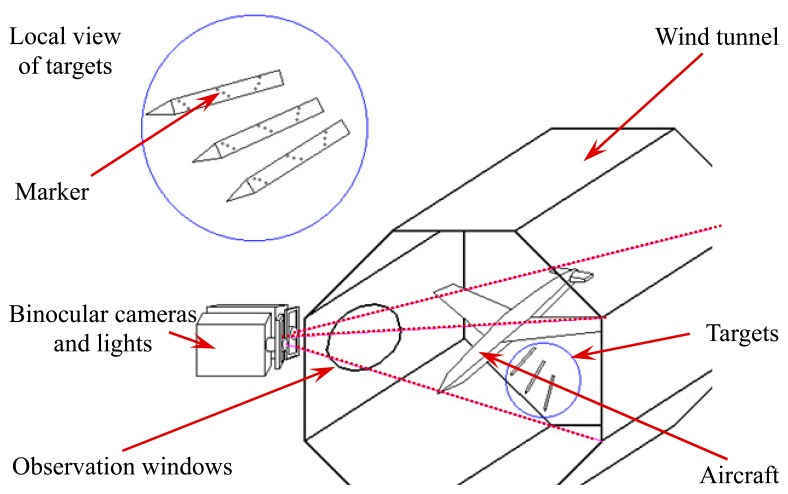
Camera-tunnel arrangement.

In this paper, position and attitude parameters of high-speed and rolling targets can be achieved *via* analyzing image sequences of markers' moving trajectory which is captured in real time by a binocular vision measurement system. The measurement principle for position and attitude parameters of high-speed moving targets is depicted in Figure 3. Firstly, ultrathin retro-reflection markers are attached on the surface of target. Then, the left and right CCD cameras are used to capture the image sequences by applying the high-speed image acquisition technique. Moreover, the high-precision matching and recognition of the corresponding markers in the left and right images are facilitated based on spatial encoding, and then the 3D coordinates of the markers in the world coordinate system are reconstructed. Furthermore, the axis of the slender rolling target is fitted using Case Deletion Diagnostics. Finally, the pose information of the target is obtained by computing the 3D coordinates of the markers.

### 2.3. Ultrathin Retro-Reflection Markers for High-Speed Image Acquisition

The high speed image acquisition of rolling targets in a wind tunnel mainly has the following difficulties: (a) Owing to the limited size of the observation window and the high image acquisition speed, it is difficult to guarantee clear images; (b) Since the target is mostly a small-sized cylinder-like object of complicated shape, it is difficult to ensure the position accuracy of markers; (c) In order to guarantee the aerodynamic shape of the scaled model, the thickness of markers cannot exceed 30 um. However, normal markers are too thick (160 μm) to meet the requirements. To solve the aforementioned problems, an ultrathin retro-reflection markers fabrication technique based on a rolling-printed method is proposed to allow the high speed image acquisition of high-speed rolling target. Ultrathin retro-reflection markers are made by mixing glass microbeads, silver powder and resin according to the specific mixing ratio of 16:5:9. The thickness of the resulting ultrathin retro-reflection markers is only 20 μm, which guarantees the aerodynamic shape of the scaled model.

**Figure 4.**
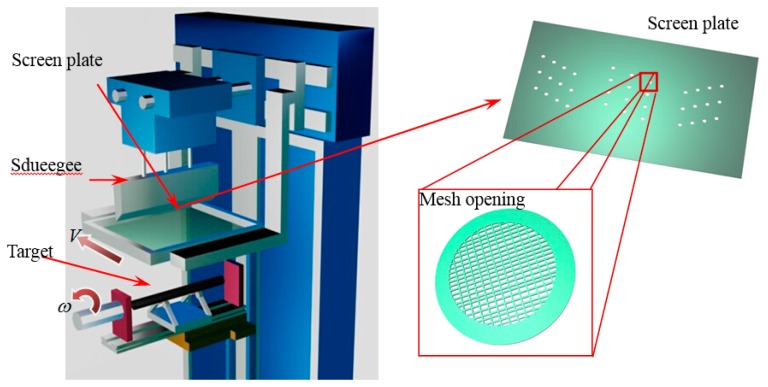
Diagram of the rolling-printed method and structure of the screen plate.

**Figure 5.**
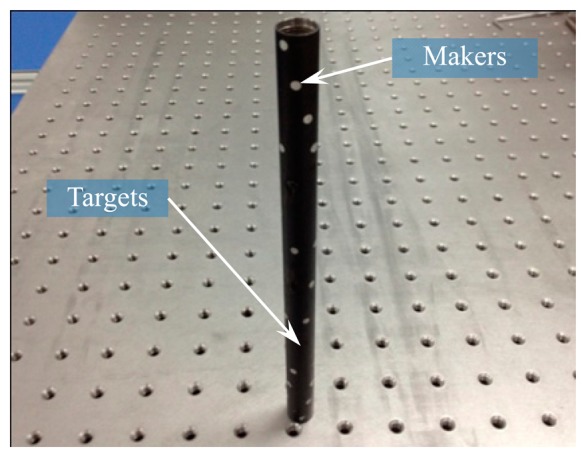
The finished target.

The diagram of the rolling-printed method is shown in Figure 4, where the screen plate that is designed according to the marker layout is placed atop the target. The ultrathin retro-reflection material is placed on top of the screen plate. When a process performs an operation on the device, the screen plate keeps in pure rolling contact with the target and moves continuously at a certain linear speed *V* along the peripheral velocity direction. *V* can be computed by *V* = *ωr*, where *ω* and *r* depict the angular velocity and radius of the rolling target, respectively. As a result, the ultrathin retro-reflection material in the mesh opening can be pumped or squeezed by the squeegee on the surface of the target. Figure 5 presents the finished target.

**Figure 6.**
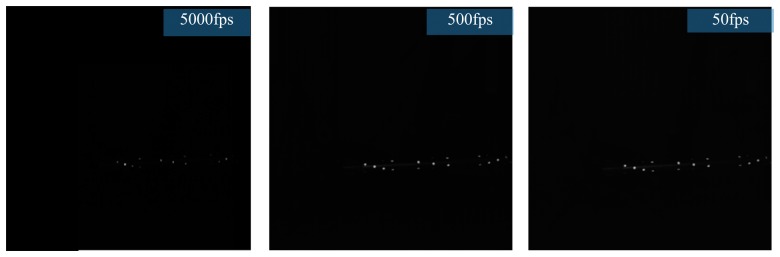
Shooting picture of ultrathin retro-reflection markers in different frames.

In this way, the thickness and the positioning accuracy of markers are ensured. Besides, images captured by the left and right cameras have higher signal-to-noise ratio and better brightness, and the image acquisition requirements can be satisfied under high shooting frequency conditions. As shown in Figure 6, the brightness of markers decreases while the shutter speed increases. However, markers are brighter and have higher signal-to-noise ratio at the 5000 shooting speed.

## 3. Image Processing

In this paper, cooperating markers are adopted to achieve rapid pose calculation of rolling targets. In order to improve the stability and precision of the results, it is necessary to attach more markers on the surface of the target. However, due to the small imaging area and lack of feature information, it is hard to match and recognize these markers. This section describes how to match and recognize markers with information loss.

### 3.1. Markers Layout of High-Speed Rolling Target

In order to calculate the pose parameters of the moving target quickly and accurately, the following problems must be solved: (1) it is difficult to acquire accurate matching and recognition of markers with less imaging area and inconspicuous motion features; (2) the high-speed rolling target in this paper is long and thin, making the target surface really small. Consequently, marker distribution is relatively dense, so that the recognition and matching of large number of markers would be very difficult; (3) markers' features (quantity, serial number, layout, *etc.*) are also difficult to be ascertained during the measuring process owing to the rolling motion of the target. To solve those problems, a spatial encoding method is presented in this paper.

**Figure 7.**
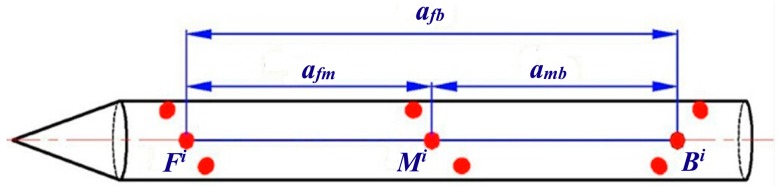
Markers distributed along the circumferential direction of the target.

Traditional encoding methods complete the encoding process by changing the shape of a single marker. Combining with dividing several markers into one group based on spatial relation, positional relationship between markers in the same group is used in the spatial encoding method proposed in this paper to realize encoding process [15]. As a result, the problem that small markers in larger field of view are difficult to encode is solved. As shown in Figure 7, each three markers are divided into one group which is called a spatial encoding group according to a linear constraint, and then the three markers can be ascertained through the distance ratio between them. The formula can be given by:

(3) $$\frac{{a^{i}}_{fm}}{{a^{i}}_{mb}}=c_{i},\frac{{a^{i}}_{fm}}{{a^{i}}_{mb}}\neq \frac{{a^{j}}_{fm}}{{a^{j}}_{mb}}$$

(4) $$\frac{{a^{i}}_{fm}}{{a^{i}}_{fb}}=l_{i},\frac{{a^{i}}_{mb}}{{a^{i}}_{fb}}=n_{i},\frac{{a^{i}}_{fm}}{{a^{i}}_{fb}}\neq \frac{{a^{i}}_{mb}}{{a^{i}}_{fb}}$$

where *a^i^* represents distance between the two space encoding markers in the same group of *i*; *f*, *m* and *d* stand for head, middle, tail part of the target, respectively; let *a^i^_fm_* be the distance between two markers in the head and middle part of the target; *l_i_* is the distance ratio between *a^i^_fm_* and *a^i^_fb_*, which can distinguish the *i*th group from all other groups; *c_i_* is the distance ratio between *a^i^_fm_* and *a^i^_mb_*; *n_i_* is the distance ratio between *a^i^_mb_* and *a^i^_fb_*; in this paper, we defined *V* be encoding array, then the encoding array of group *i* can be written as *V_i_* = [*l_i_c_i_n_i_*], and the spatial encoding of markers can be accomplished by making the array elements in every encoding array unique.

**Figure 8.**
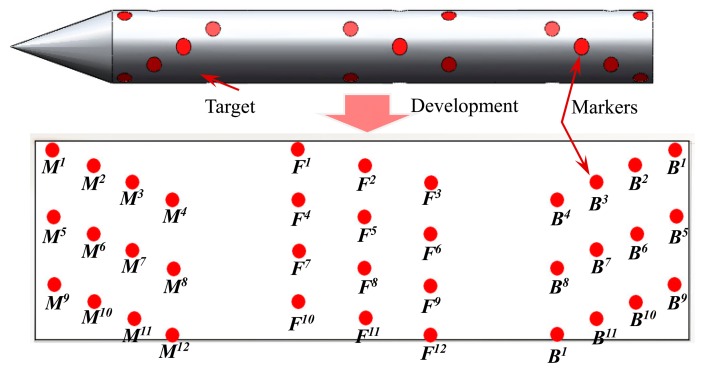
The markers layout.

To improve the precision and stability of the measurement system, it is necessary to increase the number of markers in the layout to satisfy the extraction precision. Moreover, a radial circular uniform distribution is adopted to solve the problem of the rolling motion of the target. To meet the requirements of image acquisition, a total of 12 spatial encoding groups are attached onto the target accurately according to the distance information of these three markers, as shown in Figure 8.

In the process of measuring the pose of the target, at least three groups with a total of nine markers appear in the image simultaneously. The well-proportioned distribution of markers in the head, middle, and tail part of the target guarantees the number of markers as well as the pose estimation precision of the target.

### 3.2. Feature Extraction

In the complicated wind tunnel conditions, it is difficult to conduct the marker feature extraction due to such factors as small-sized model with large curvature change, large field of view and small imaging area of markers. Therefore, the gradient weighted method is employed to extract the center of the markers with high accuracy. Firstly, a convolution operation of the image is conducted based on a Gaussian first-order differential operator in order to obtain the gradient of each point in the image. The Gaussian first-order differential operator can be written as:

(5) $$h^{\prime }(x)=\frac{-x}{\sqrt{2\pi \sigma }}e^{-\frac{x^{2}}{2\sigma^{2}}}$$

Then, the gradient of image can be given by:

(6) $$\left|G\left(x,y\right)\right|=\left[\begin{array}{c} F_{x}^{\prime }\\ F_{y}^{\prime } \end{array}\right]=\left[\begin{array}{c} F\left(x,y\right)⊗h_{x}^{\prime }\\ F\left(x,y\right)⊗h_{y}^{\prime } \end{array}\right]$$

The coordinates of gradient centroid of markers pattern can be expressed as:

(7) $$c=\sum_{i=-h}^{h}\sum_{j=-w}^{w}\left[\left|G\left(i,j\right)\right|\cdot P\left(i,j\right)/\sum_{i=-h}^{h}\sum_{j=-w}^{w}G\left(i,j\right)\right]$$

where *c* is the coordinates of the gradient centroid of the markers; |*G*(*i*,*j*)| represents the weight of point (*i*,*j*); *w* and *h* stand for the width and height of target image, respectively; *P*(*i*,*j*) is the image coordinate of the point (*i*,*j*).

### 3.3. Feature Matching between Two Views

Gray matching and feature matching are generally used for the classical and effective image matching. The gray matching method is a mathematical procedure based on the gray information and image technique, while features such as color, texture, shape, spatial position extracted from two or more images are used in feature matching. However, high-precision matching under extreme environment conditions cannot be achieved according to the gray and feature information due to the fact that markers captured by ultra high-speed cameras have a small area and the gray similarity between each one is big.

**Figure 9.**
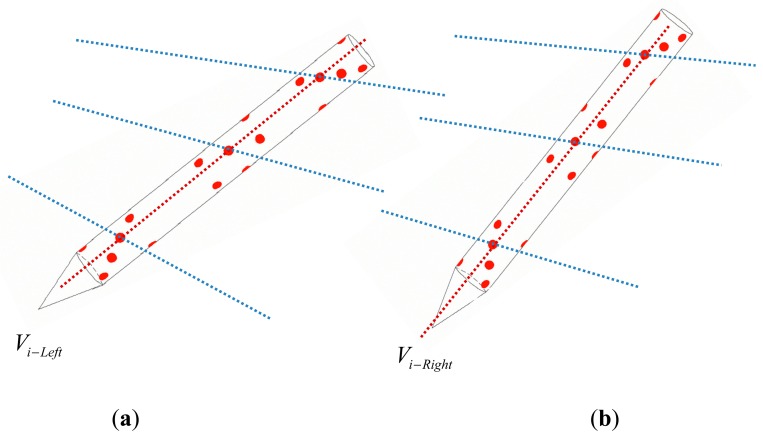
Matching between left and right images.

To solve the problem, spatial encoding markers are applied to transform the traditional matching method based on features into a problem according to spatial constraints between markers, as shown in Figure 9.

Firstly, spatial encoding markers in different groups are isolated by a collinear constraint, and then the matching relationship between the left and right images can be obtained through comparing *V_i_*_–_*_Left_* (obtained in Section 3.1) of the right image and *V_i_*_–_*_Right_* of left image. Moreover, based on the epipolar constraints, the corresponding marker matching is verified and the mismatched markers are removed.

### 3.4. The Recognition Method

**Figure 10.**
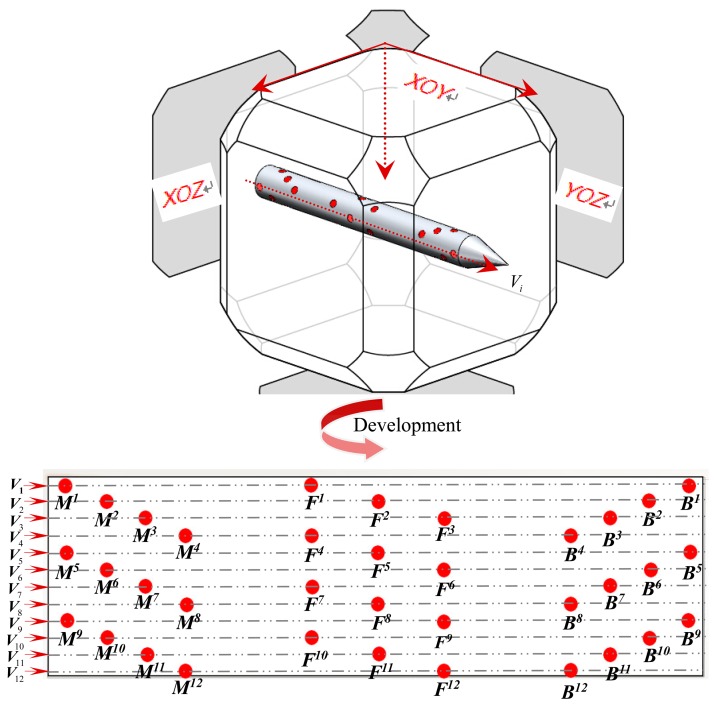
Spatial model of marker recognition.

The recognition process can be described as follows: (a) Reconstructed markers are grouped according to Section 3.3; (b) Recognition relationships can be obtained by comparing in turn each encoding array *V* obtained by construction (Section 3.1) with the one *V_i_* (*i*=1,2…12) determined by markers layout, as shown in Figure 10.

## 4. Position and Attitude Solution

The position and attitude parameters can be obtained by translating the coordinates from the local coordinate system on the slender rolling target to the world coordinate system through the translation and rotation matrix. Firstly, the axis of the slender rolling target is fitted by Case Deletion Diagnostics. Then the coordinates of the centroid can be solved based on the distance and axis constraint. Moreover, the absolute position method is applied to calculate the initial translation and rotation coordinate system which are used for the transformation, and at last the position and attitude information can be obtained.

### 4.1. Axis Fitting Based on Case Deletion Diagnostics

Errors caused by mismatching points have a great influence on the axis fitting solution. Thus, to guarantee the fitting precision, an axis fitting method based on Case Deletion Diagnostics is proposed to remove mismatched points. Case Deletion Diagnostic is commonly used to assess the influence of observations on the parameter estimators through subset deletion [16]. Utilizing the Case Deletion Diagnostic, the outlier or strong influence points are diagnosed by comparing the statistic difference between the deleted model and un-deleted model, thus the gross errors can be removed. Specifically, suppose that the data is *P*={*P*_1_,*P*_2_,…,*P*_n_}, when removing the *i*th data point, if there is a difference between the deleted model and the un-deleted model, the outlier or strong influence points can be obtained, otherwise we can acquire the correct points which have little impact on statistical results.

Let the total number of the markers be *n* and at least nine markers can be collected according to markers layout. Then the point set under the world coordinate system can be given by:

(8) $$P=\left\{P_{1},P_{2},\cdots ,P_{n}\right\}$$

(9) $$P_{i}=\left(x_{i},y_{i},z_{i}\right),\left(i=1,2,\ldots ,n\right)$$

where (*x_i_*,*y_i_*,*z_i_*) describe the 3D coordinates of point *P_i_* in the world coordinate system. *P* stands for a point set. Case Deletion Diagnostic *M_w_*(*θ*) for axis fitting can be set up as follows:

(1) The point set is applied to fit the axis equation *L* according to the theoretical radius *r* which represents the distance between markers and axis.
(2) The parameter estimators *θ* can be expressed as:
  (10) $$\theta =\sqrt{\sum_{i=1}^{n}{\left(d\left(p_{i}\right)-r\right)}^{2}}$$
  where *d*(*p_i_*) is the euclidean distance from point *p_i_* to axis *L*; axis *L̂* for slender rolling target can be obtained after deleting the point *p_w_*.
(3) The estimated value *θ̂* of parameter estimators can be given by:
  (11) $$\hat{\theta }=\sqrt{\sum_{i=1,i\neq w}^{n}{\left(\hat{d}\left(p_{i}\right)-r\right)}^{2}}$$

The judgment that whether the point *p_w_* is outlier or strong influence point can be expressed as:

(12) $$\left|\hat{\theta }-\theta \right|\leq \varepsilon$$

where *ε* represents the threshold defined above. When the above inequality is satisfied, we can get the result that point *p_w_* is a normal point, otherwise it is a strong influence point. The initial point set for Case Deletion Diagnostic *M_w_*(*θ*) is all the markers collected. Every point is judged one by one, and a point within the set can be removed if it is outlier or strong influence point. Afterwards, the axis can be solved stably using the LM optimizing algorithm:

(13) $$\{\begin{array}{l} \min\sqrt{\overset{n}{\underset{i=1}{\text{∑}}}{\left(d\left(p_{i}\right)-r\right)}^{2}}\\ \begin{array}{cc} s.t. & \frac{x-x_{0}}{m}=\frac{y-y_{0}}{n}=\frac{z-z_{0}}{p} \end{array}\\ \begin{array}{cc} & d\left(p_{i}\right)=\frac{\sqrt{\left|\begin{array}{cc} y_{i}-y_{0} & z_{i}-z_{0}\\ n & p \end{array}\right|+\left|\begin{array}{cc} z_{i}-z_{0} & z_{i}-x_{0}\\ p & m \end{array}\right|+\left|\begin{array}{cc} x_{i}-x_{0} & y_{i}-y_{0}\\ m & n \end{array}\right|}}{\sqrt{m^{2}+n^{2}+p^{2}}} \end{array} \end{array}$$

where *P*_0_(*x*_0_,*y*_0_,*z*_0_) stands for one point on the fitted axis; *L*(*m*,*n*,*p*) is the direction vector of axis; *d*(*p_i_*) represents the distance between point *P_i_*(*x_i_*,*y_i_*,*z_i_*) and the fitted axis.

### 4.2. Solution for Centroid Position Based on Distance Constraint

The centroid coordinates can be solved based on the distance constraint between markers. In this paper, about nine to twelve markers can be extracted according to the layout design. In order to improve the centroid coordinates' precision, optimization based on the distance between all markers captured and centroid are carried out. However, the aforementioned distance is different, and the extracted markers have different degrees of brightness and circularity. These factors have a different impact on the precision of the centroid coordinates. Thus, multi-points optimization based on setting weight for every point is presented. Since the centroid is on the axis, the centroid position can be optimized according to axis position calculated *via* Section 4.1. The optimization formula can be given by:

(14) $$\left\{ \begin{array}{l} \min\left(\overset{n}{\underset{i=1}{\text{∑}}}\rho_{i}{\left(Dc_{i}-dc_{i}\right)}^{2}\right)\\ \begin{array}{cc} s.t. & \frac{x_{c}-x_{0}}{m}=\frac{y_{c}-y_{0}}{n}=\frac{z_{c}-z_{0}}{p} \end{array} \end{array}\right.$$

where *ρ_i_* represents the weight value of each captured marker; *Dc_i_* stands for the measured distance between the centroid and the optimized markers; *dc_i_* is the real distance between the centroid and optimized markers; *C*(*x_c_*,*y_c_*,*z_c_*) is the centroid coordinate; *L*(*m*,*n*,*p*) stands for the direction vector of the axis.

### 4.3. Absolute Pose Solution

In this paper, a pose acquisition method is carried out according to the aforementioned marker layout. Two coordinate systems−the initial reference coordinate system (IRCS) and the local coordinate system (LRCS), are established to acquire the pose information of the target through coordinate transformation. The IRCS represents the reference coordinate system set up at the first frame, while the LRCS means the local coordinate system set up at the frame of the measuring position.

**Figure 11.**
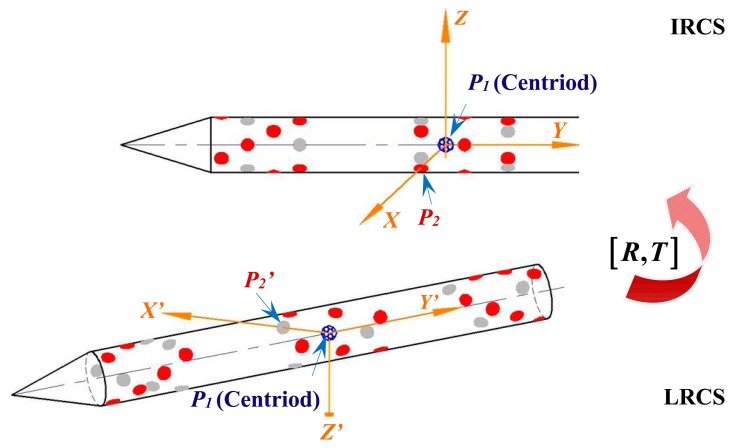
Schematic of the principal of absolute pose solution.

As shown in Figure 11, when the axis fitting is finished (Section 4.1) and the centroid position determined (Section 4.2), the centroid of target *P*_1_ is set as the origin of the reference coordinate system and the axis of the target is defined as the Y axis of the reference coordinate system.

Moreover, since the X axis can be defined by any marker captured by cameras with high image quality, the optimum marker *P*_2_ is chosen as the definition point. As a result, the space line through the centroid of the target parallel to the line through *P*_2_ and perpendicular to the rotation axis of the target is defined as the X axis. Then, according to the right-handed coordinate system, the reference coordinate system can be set up.

To obtain the pose parameters, it is necessary to establish local coordinate system using the same marker *P*_2_. However, marker *P*_2_ may not be captured at the position measuring frame owing to the hidden markers phenomenon caused by the rolling movement. In order to establish the local coordinate system, the three-dimensional coordinates of all visible makers at the frame of the measuring position are reconstructed. Then, based on the recognition of visible markers and markers layout, the three-dimensional coordinates of the center of marker *P*_2_ can be acquired. Afterwards, the local coordinate system can be obtained using the same establishment principle for the reference coordinate system.

The relationship between the point *p* = (*x y z*)*^T^* in IRCS and the corresponding *p′* = (*x′ y′ z′*)*^T^* in LRCS can be written by:

(15) $$\left[\begin{array}{c} x'\\ y'\\ z' \end{array}\right]=R_{pt}\left[\begin{array}{c} x-x_{0}\\ y-y_{0}\\ z-z_{0} \end{array}\right]$$

where (*x*_0_ *y*_0_ *z*_0_) is the coordinates of the origin of the target in IRCS. Moreover:

(16) $$R\left[\begin{array}{c} x\\ y\\ z \end{array}\right]+T=\left[\begin{array}{c} x'\\ y'\\ z' \end{array}\right]$$

where:

(17) $$\begin{array}{l} R=R_{\theta_{Y}}R_{\theta_{X}}R_{\theta_{Z}}\\ =\left[\begin{array}{ccc} \cos(\theta_{Y})\cos(\theta_{Z})-\sin(\theta_{Y})\sin(\theta_{X})\sin(\theta_{Z}) & \cos(\theta_{Y})\sin(\theta_{Z})+\sin(\theta_{Y})\sin(\theta_{X})\cos(\theta_{Z}) & -\sin(\theta_{Y})\cos(\theta_{X})\\ -\cos(\theta_{X})\sin(\theta_{Z}) & \cos(\theta_{X})\cos(\theta_{Z}) & \sin(\theta_{X})\\ \sin(\theta_{Y})\cos(\theta_{Z})+\cos(\theta_{Y})\sin(\theta_{X})\sin(\theta_{Z}) & \sin(\theta_{Y})\sin(\theta_{Z})-\cos(\theta_{Y})\sin(\theta_{X})\cos(\theta_{Z}) & \cos(\theta_{Y})\cos(\theta_{X}) \end{array}\right] \end{array}$$

As a result, the centroid position of the target in the world coordinate system can be represented by *T_pb_*= (*x*_0_ *y*_0_ *z*_0_); –*θ_Z_*, –*θ_X_*, –*θ_Y_* represent the three attitude parameters of the target in the world coordinate system: the yaw, pitch and roll. According to the coordinates of the chosen points mentioned above, the position and attitude parameters of the target can be computed.

## 5. Experimental Analysis

Experiments on pose measurement of the target in a wind tunnel are conducted in this paper, as well as the precision verification test. In this section, the measurement precision of the three directions and three angles is verified.

### 5.1. Vision Measurement System

**Figure 12.**
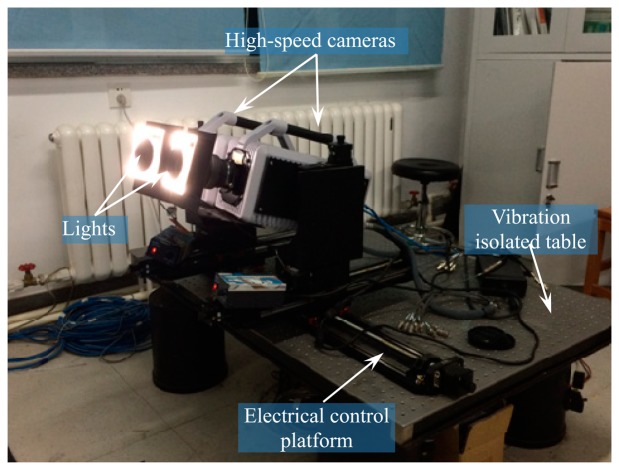
Mechanical support and hardware for cameras.

A wind tunnel is a dark narrow enclosure which can only be monitored by an observation window with a fixed shape. According to the actual wind tunnel structures, an ultra high-speed binocular vision measurement system is set up, as shown in Figure 12. The binocular vision measurement system contains two high-speed cameras (FASTCAM SA-X), two wide-angle lenses (Nikon 1735), two low angle lights, a shockproof platform, two electronic control platforms and a processing terminal. To minimize the influence of vibrations caused by the wind tunnel at runtime, the two cameras are placed symmetrically on a customized air flotation shockproof table. The camera system is installed on the electric control platform so that it can be translated and rotated as designed. The low angle DC lights are installed on the edge of the lenses to ensure the light requirements of images are met.

### 5.2. Measurement Experiment for Rotary Target in Wind Tunnel

**Figure 13.**
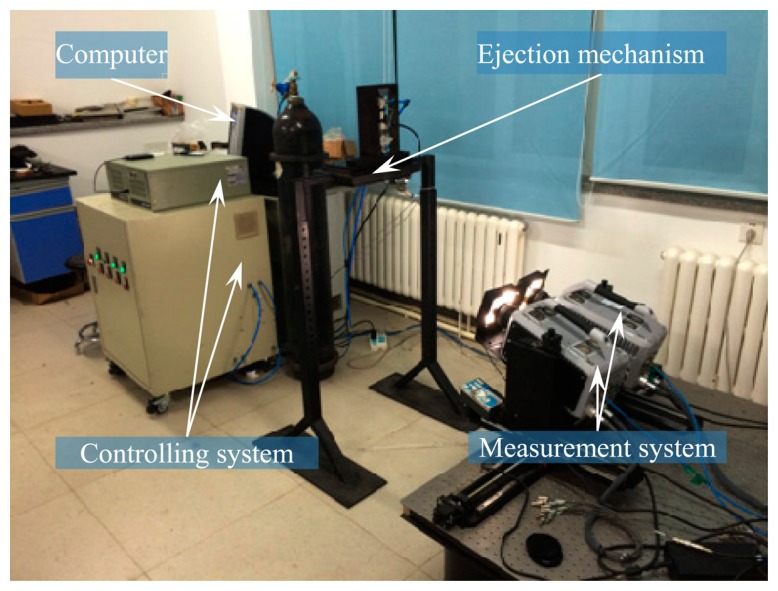
Layout of experimental facilities.

As shown in Figure 13, the experimental facilities consist of a pose measurement system based on binocular vision, an ejection mechanism and a controlling system.

In the test with measurement field of 1 m × 1 m and measurement rate of 5000 fps, the ejection mechanism and controlling system are utilized to eject the target out of the tank with a particular velocity, angular velocity and angle. The vision measurement system is triggered to measure the pose information of the target synchronously. Based on the aforementioned method, the pose measurement resulte of the rolling target are verified.

**Figure 14.**
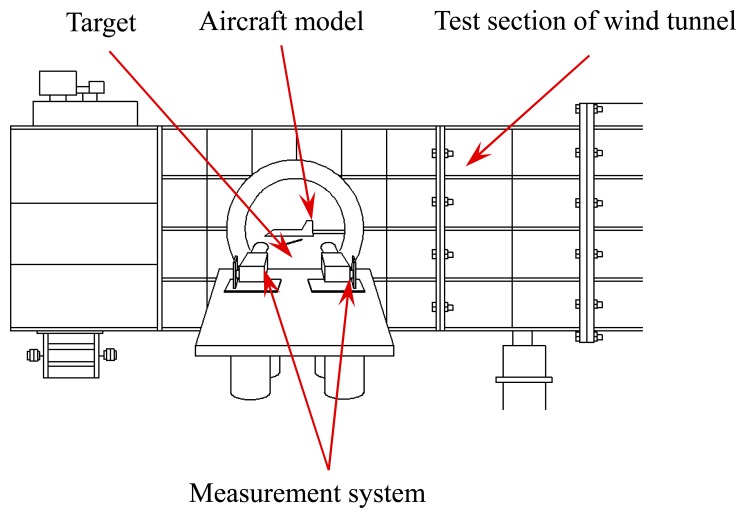
Facility layout for the wind tunnel test.

To calculate the actual pose parameters of the target, experiments were conducted in a closed supersonic wind tunnel (FL-3). The facility layout for the wind tunnel tests is shown in Figure 14.

Specifically, an aircraft model is installed in the inner wall of the test section, and the ejection mechanism is installed in the ejection cabin of the aircraft model. Besides, the measurement system is arranged outside of the test section to measure the pose parameters of the object in real time through the observation window.

**Figure 15.**
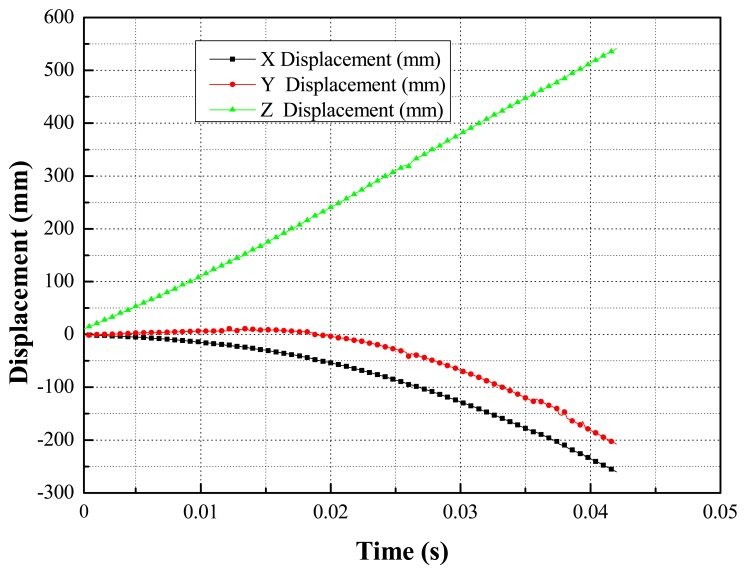
The results of the position measurement in the wind tunnel.

**Figure 16.**
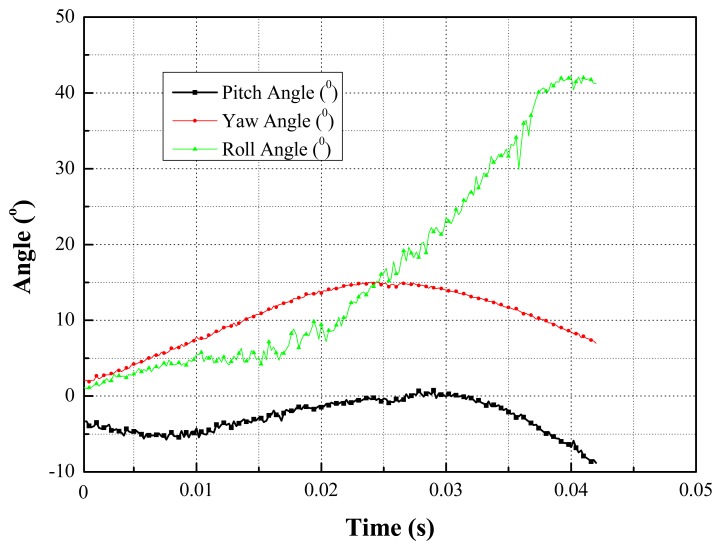
The results of the attitude measurement in wind tunnel.

Quick pose measurements of the high-speed and rolling target with different inertia parameters under different Mach wind speeds are conducted. For instance, tests under 0.2 Ma, 0.4 Ma and 1.5 Ma, are completed in the transonic wind tunnel. Afterwards, the pose parameters can be given in the wind tunnel coordinate system, in which the *X*-axis and *Y*-axis are parallel to the airflow direction and the direction of gravity, respectively. Additionally, one point on the airplane cabin door is selected as the origin of the wind tunnel coordinate system and the *Z*-axis is established according to the right-handed system. The measured results for certain types of targets with 1.5 Mach speed are depicted in the following diagrams. The results of the position measurement are shown in Figure 15, and the results of the attitude measurement are shown in Figure 16.

Experimental results indicate that the quick intellectualized pose measurement of the target can be guaranteed by the proposed method and established measurement system. According to the experimental analysis, three main factors affect the pose measurement accuracy: (a) Problems such as large measuring range, limitation of the resolution of the ultra-high speed camera directly affect pose measurement precision of the target; (b) The extraction accuracy of the markers is affected due to factors such as small volume, surface profile of markers and random deformation during the rolling process.

### 5.3. Precision Verification Test in Laboratory

In this paper, targets are fixed on a high precision motion control stage. The *X*, *Y*, *Z* axis position measurement precision is verified through shooting the movement of targets when the motion control stage moves a fixed distance along the *X*, *Y*, *Z* axis direction with high precision. Then the attitude measurement precision of the measurement system is verified with the target rotating around the *X*, *Y*, *Z* axis with a fixed angle.

**Figure 17.**
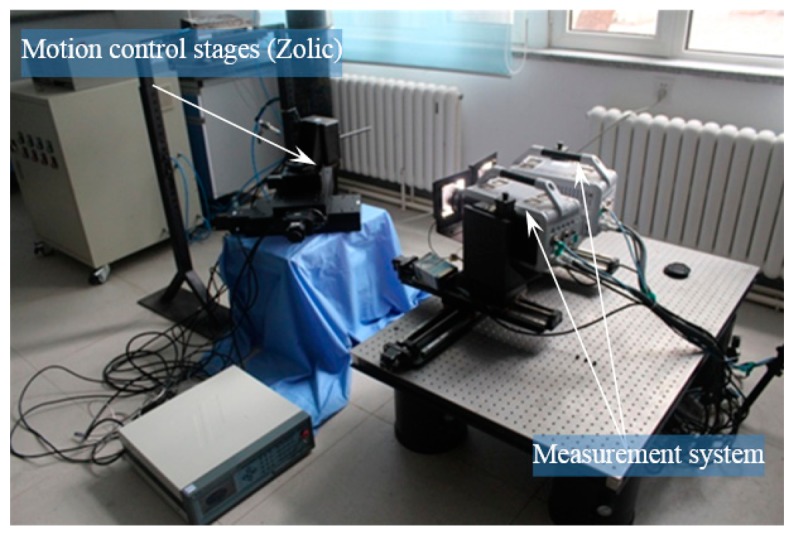
The calibration diagram of measurement system.

**Figure 18.**
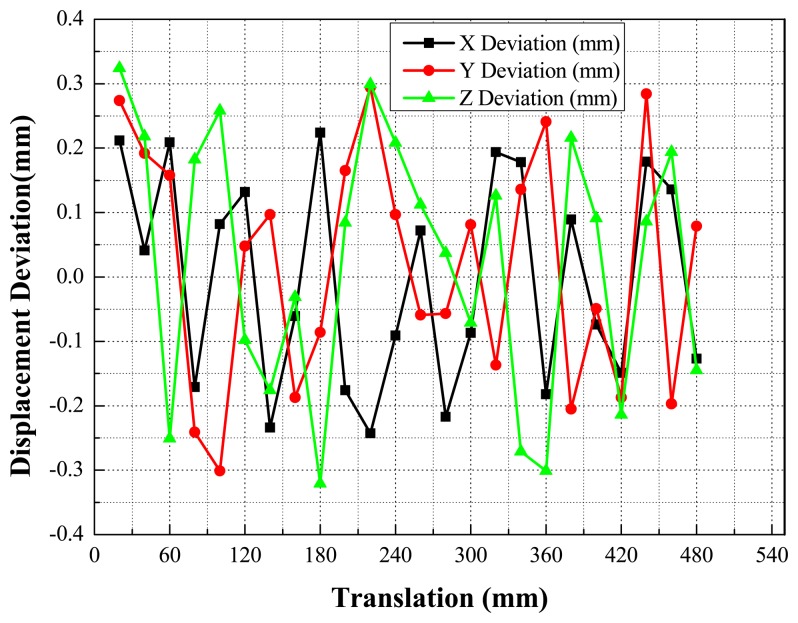
The position measurement precision.

**Figure 19.**
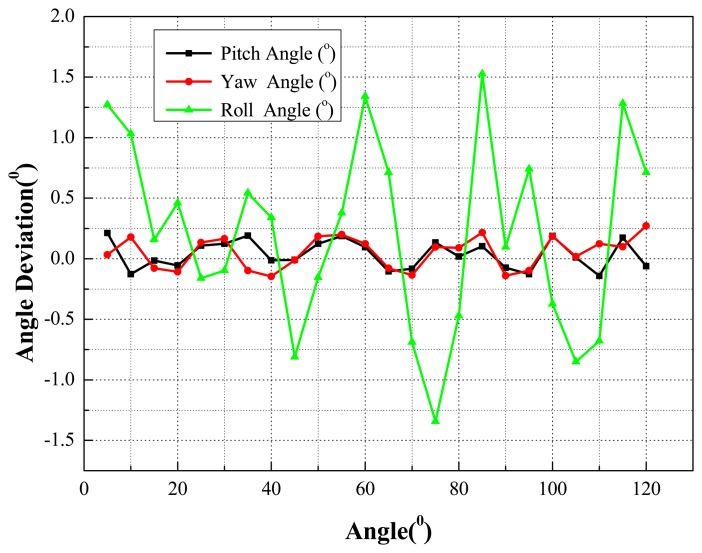
The angle measurement precision.

The experimental facilities are shown as Figure 17. For each camera, a high precision motion control stage combining a displacement platform and a rotary platform is built. With a position precision of 0.001 mm, the motion control stage is used to implement the specified moving distance of targets. Twenty four displacement accuracy verification test runs were conducted, and the target moved 20 mm in each run. Twenty four angle accuracy verification test runs were also conducted, where the target rotated five degrees around the three axes, respectively, in each run. The displacement measurement precision in each run is depicted in Figure 18. The measured angle precision in each run is shown Figure 19.

As shown in Figures 18 and 19, the mean square error of the displacement measurement in the X direction is 0.13 mm, while in the Y direction it is 0.14 mm, and in the Z direction 0.16 mm. The main errors mainly come from errors in the markers' center extraction and camera calibration. The mean square error of the pitching angle precision is 0.115°, and that of the yaw angle precision 0.132°. The high-precision solution of the measurement system for pitching angle and yaw angle results from the high-precision of axis fitting. The roll angle precision is 0.712°, mainly due to the small diameter of the object to be tested. In conclusion, the experimental results indicate that the proposed method can exhibit high accuracy for the position and attitude measurement of high-speed and rolling targets in a wind tunnel.

## 6. Conclusions

In this paper, we have presented a pose measurement method for wind tunnel tests to measure high-speed and rolling targets. A high-speed image sequence acquisition method based on ultrathin retro-reflection markers is presented to ensure high brightness and SNR. Ultrathin retro-reflection markers attached on the surface of a target by a rolling-printed method is as thin as 20 μm, guaranteeing the aerodynamic shape of the scaled model.

A novel marker layout on the basis of spatial coding is proposed to ensure that markers are distributed evenly on the target surface and to achieve high-accuracy pose information. This layout overcomes the problem of the information loss of markers when the target is rolling and ensures matching, so that accurate marker recognition can be obtained.

Moreover, a pose parameter computing method is proposed according to the markers' 3D coordinates. Besides, an axis fitting method based on Case Deletion Diagnostic can increase the stability of the measurement system and reduce the errors caused by mismatching.

The experimental results described above indicate that the proposed pose measurement system and method can achieve high accuracy and stability, and meets the requirements of wind tunnel tests. Finally, the rapid pose measurement with high precision for rolling targets is accomplished in a transonic wind tunnel environment, and good results are achieved. Our future research will focus on improving the pose measurement precision by algorithm optimization.
